# Pathological Society of London

**Published:** 1847-03

**Authors:** 


### PATHOLOGICAL SOCIETY OF LONDON.

Mr. Ebenezer Smith exhibited a fœtal heart, presenting *Premature Occlusion of the Foramen Ovale; diseased Mitral Valve; contracted left Heart; large Pulmonary Artery and Duct.*

The preparation was accompanied with diagrams. The following is the history connected with the case:—

A small infant, the seventh of healthy children by the same parents, was born on Oct. 20th, 1846. His mother had noticed unnatural quietude during the last three months of gestation. Its birth was natural, the head, body, and limbs, well developed, and of the usual colour, till, in about five minutes after it first breathed, its face became dark blue, and the respiration difficult. The ligature on the umbilical cord was untied, but no blood flowed; the skin soon became similarly blue; and the respiration very difficult and irregular; the heart beat about 130 in a minute, clearly and strongly; the temperature appeared natural. In eight or ten hours apoplexy came on, the cyanosis being universal, the right arm paralyzed, and the left convulsively contracted. In sixteen hours the child was perfectly comatose, and it died twenty-one hours after birth.

*Inspection, twenty-four hours after death.*—The body full grown, well proportioned; skin marbled with large dark and white patches; pericardium contained two or three drachms of serum; the lungs about the usual size, full of dark blood, slightly emphysematous anteriorly; the liver apparently healthy; umbilical vein pervious and empty; venæ cavæ capacious; heart rather more vertical than natural, smaller and narrower in consequence of flatness of the left ventricle; both venæ cavæ, the right heart, and coronary veins, engorged. On the opening of the right auricle, which was distended, and of the usual form, the fossa ovali was seen in its natural position, but closed by a strong reticulated membrane firmly attached to its distinct annulus, impervious and pouched. A probe could be passed down to the left three lines into a considerable impervious inter-auricular fossa. Below the fossa ovalis, a mere vestige of the Eustachian valve; valves of the coronary vein distinct; the right auriculo-ventricular opening large, patent, and furnished with a very good valve, differing in form from the usual shape of the tricuspid, but with distinct, fixed and distended columns and curtains, and moderator bands, as demonstrated by Mr. Wilkinson King; walls of the right ventricle rather hypertrophied, the cavity being large, and not only descending to the apex, but curving round the left ventricle, so as alone to form the apex of the heart: no inter-ventricular communication whatever. The pulmonary artery very large, and three and a half lines wide at its origin; a little above its origin, more than four lines; its valves being perfect; it gave off, in the usual situation, branches to the right and left lung, and was then continued perpendicularly upwards, in a scarcely diminished trunk, the ductus arteriosus to the under surface of the aortic arch, nearly opposite the left subclavian, so that the blood from it would flow, not only into the descending aorta, but upwards, and somewhat contrary to its previous direction, into the arteries of the head and arms. The four pulmonary veins entered the left auricle, as usual. This cavity was well developed, being wide transversely, and constricted vertically, having a very large muscular appendage. The complete membrane covering the fossa ovalis was here also quite distinct and impervious, and could be readily pushed towards the right auricle ; the auriculo-ventricular opening extremely contracted, being but one line and a half in diameter. The mitral valve was altogether defective in structure, and seemed to have been faulty in action ; it consisted of two very small, whitish, tendinous bands, fixed, the one to the septum, in front, the other to the posterior wall of the ventricle, without any curtains or fleshy bands, which, it seemed, could not have been opposed so as to prevent the reflux of the blood into the auricle. The left ventricle almost obliterated ; its walls contracted on a small cavity at the base, not exceeding two or three lines in diameter, lined by a very dense, smooth membrane. The aortic opening also much contracted, being about two lines wide ; the sigmoid valves perfect ; the aorta itself normal in its course, giving off normal branches, but the ascending and transverse portion of the arch considerably smaller than the pulmonary artery, measuring, at its origin, three lines in diameter, and opposite the left carotid, just before receiving the arterial duct, two plies and a quarter ; the descending aorta, after receiving the arterial duct, measuring two lines and three-quarters. The arterial duct continued almost perpendicularly upwards into a large trunk, to nearly opposite the left subclavian.

Thus, during the life of the child "in utero," the systemic circulation was maintained by the increased development and perfect mechanism of the right heart and vessels, notwithstanding the useless mitral valve, the arrested development of the left heart and aorta, and the strongly closed foramen ovale. On the assumption, however, by the lungs, of their proper action, the quantity of blood received by them was obstructed in its return by the defective mitral valve, &c., respiration became embarrassed, the free circulation of blood in the brain impeded, and death from apoplexy resulted. Mr. Smith remarked that two points of especial interest arose out of this example of malformation of the heart. The first was,—Which was the primary cause? Did the premature closure of the foramen ovale increase the activity of the right heart and vessels so as to carry on the circulation of the system, and arrest the development of the left heart, and particularly the mitral valve? Or, was the diseased action first manifested in the mitral valve, which, producing an utterly defective valvular structure, stayed the blood in its course through the left heart, and presuming the valve of Botal to be early and well developed, thereby pressed that valve against its margin, so as to cause its firm occlusion ?

Judging from what appeared to be merely the vertigiform structure of the mitral valve, and from indications that the left auricle had once been more capacious, and had subsequently become constricted, Mr. Smith inferred that about the fifth month the mitral valve became inadequate to the systemic heart of the growing fœtus, obstruction and embarrassment followed, so as to produce astasis of blood in the left cavities; increased energy, perhaps inflammation, of these structures ensued, which resulted in a closure of the foramen ovale. The ductus arteriosus being open, the energies of the right heart produced so perfect a development of its parts and connected vessels, as to maintain the systemic circulation; and hence, the left heart and ascending aorta being comparatively useless, contracted in their size. This view of the state of the left heart, exclusive, however, of the condition of the mitral valve, he thought was consistent with the other theory,—that premature closure of the foramen ovale was the first link in the chain of morbid occurrences.

The second point, as urged by Dr. Norman Chevers, "That a closed foramen ovale is not a proof in jurisprudence that a child has lived;" for in this case the occlusion must have existed long before birth. The preparation is deposited in the museum of Guy's Hospital.

Dr. Peacock exhibited three specimens of *Malformation of the Heart.*

In the first case, there existed extreme contraction of the orifice of the pulmonary artery, with a deficiency in the interventricular septum, and the aorta arose in chief part from the right ventricle. The right auricle and ventricle were of large size, and the walls of the latter thick and very firm. The left ventricle was, on the contrary, small, and its walls thin and flaccid. The left auricle was also small. The foramen ovale and ductus arteriosus were both closed. The heart was taken from a child two years and five months old, who had exhibited well marked symptoms of cyanosis, which commenced three months after birth. Dr. Peacock remarked that though the cases are numerous in which, with more or less contraction of the orifice of the pulmonary artery, the septum of the ventricles was deficient, it is far from frequent to meet with these malformations, with, as in this case, a closed state of the foramen ovale; and especially so when there is great contraction of the pulmonary artery.

The second case was one in which the only departure from healthy conformation of the heart consisted in the small size of the arteries distributed to the lungs; in consequence of which, the trunk and orifice of the pulmonary artery and the right ventricle were unusually large, and the foramen ovale imperfectly closed; the left ventricle and aorta being small. The heart was taken from a child of ten weeks, which had displayed slight cyanosis during life.

The third specimen was a heart taken from a girl of ten or eleven years of age, with whose history Dr. Peacock was unacqaainted. The interesting points in this case were, first, a deficiency of the base of the inter-auricular septum, with a perfect closure of the foramen ovale. The deficient space allowed of free communication between the two auricles, so that there could only be said to be one auriculo-ventricular aperture. 2ndly. A distinctly tricuspid form of the left auriculoventricular valve ; and 3rdly, a deficiency at the base of the septum of the ventricles, closed by an extension of the anterior fold of the left auriculo-ventricular valve, and the subsequent expansion of this membrane into a small sac, having an aperture communicating with the cavity of the right auricle. Dr. Peacock suggested, that had this patient survived a few years longer, the sac and the opening into the auricles would doubtless have enlarged, and have assumed, both in the symptoms during life, and the appearances after death, a veıy close resemblance to a true aneurism of the undefended space of the base of the ventricular septum, which had opened into the right auricle.

Mr. Crisp exhibited two specimens of *Diseased Heart.*

The first Mr. Crisp believed to be one of "concentric hypertrophy of the left ventricle." The specimen was taken from a woman aged seventy-nine. She was only seen twice before death, and then appeared to be in great pain, but owing to mental imbecility, was unable to describe her symptoms.

Nothing remarkable was observed about the thoracic or abdominal viscera: the thoracic aorta and branches being more than usually healthy. The abdominal aorta, for three inches above its bifurcation, was converted into a firm bony cylinder. The right internal iliac nearly impervious from ossific deposit; the left less ossified: the common and internal iliacs were tolerably heathy. In the lower part of the thoracic aorta was a dense fibrinous coagulum, occupying the calibre of the vessel, but not adhering to its sides. It was composed principally of fibrin, with a few blood globules.

Case 2.—The patient from whom this heart was taken first came under Mr. Crisp's care in April, 1841, when he was fifty-six years of age. His health before this period had been tolerably good, with the exception of slight attacks of difficulty of breathing. (His father died suddenly of angina pectoris, at the age of fifty-five.) At that time he suffered from great difficulty of breathing, which came on suddenly, pain and sense of oppression at the præcordia ; pulse irregular ; heart's sounds normal, but its impulse increased ; pain in the left arm, extending to the elbow, and sometimes affecting the little and ring fingers ; he had also violent pain in the epigastrium, so as to induce him to suppose that he was passing gall-stones, although none could be discovered in the evacuations. These symptoms continued for several days ; he was bled from the arm, leeches and fomentations were applied to the side, and small doses of mercury given, so as slightly to affect the gums. He gradually improved in health, and was able to resume his usual avocations ; he suffered, however, from frequent attacks of angina pectoris, and the pain in the left arm and fingers was at times very severe, lasting occasionally for four or five days ; latterly, he had pain also in the right arm and shoulder. About eighteen months since, he suffered from acute pain in the left side, and a few days afterwards, a small calculus was voided with the urine. Of late, his health had been better than usual.

Three days before his death he complained of excruciating pain in the left arm and little finger, which was somewhat relieved by warm applications, but it soon returned. He also complained of a sense of oppression in the region of the heart—"as if (to use his own expression) the heart had been bound down." (The pain and oppression varied in severity, but continued more or less to the time of his death.) The pulse was about the same as usual, except during the acute attacks of pain, when it was quickened and less bounding. He retired to bed about half-past ten on the night previous to his death, slept for five or six hours, and in the morning expressed himself easier than he had been for two or three days. He was able to dress and shave himself, and walk down stairs; and whilst in the act of getting his breakfast, his head suddenly fell forward, and he died instantaneously.

*Examination, twenty-two hours after death.*—Body warm; blood fluid; face very pallid; great abundance of fat, about three inches in thickness, on the abdominal parietes.—*Chest:* Numerous pleuritic adhesions on the left side. Both lungs gorged with black blood, especially the left, but the blood was confined to its proper vessels. The heart was large and flabby, (weight about fourteen ounces.) Both the coronary arteries were ossified in various parts, in some places so as to form a bony cylinder; the left ventricle dilated; both auriculo-ventricular openings large; but the mitral and tricuspid valves appeared to have performed their proper functions. The aorta was of its usual calibre, but it contained numerous elevated patches of cartilaginous and atheromatous deposit, as well as three bony plates; its colour natural. Liver large, but its structure apparently healthy. The gall-bladder filled with calculi, and its duct impervious, (as in the case which was related to this Society by Mr. Crisp a few weeks since.) Right kidney double its natural size; left, small; pelvis of both congested; the right contained a small phosphatic calculus. Brain not examined.

Dr. F. H. Ramsbotham presented an *Infant's heart, possessing only one Auricle and one Ventricle.*

It was situated in its natural position in the chest. The auricle was separated from the ventricle by tendinous valves. There was only one artery, the aorta, arising from the ventricle, which sent off a vessel in the situation of the ductus arteriosus. This artery divided into two branches, which supplied the lungs. The venæ cavæ and the two right pulmonary veins entered the auricle in their ordinary positions and directions, the pulmonary veins of the left side forming one common trunk before their termination in the auricle. The vena cava inferior, however, had been destroyed by opening the auricle, and the branch passing from the aorta, which divided into the pulmonary arteries, had unfortunately been cut away in removing the heart from the body.

The child was of full time, well developed, and lived ten days. The late Dr. Combe, who attended it during its short life, stated that cyanosis was perfect over the whole body, but that neither the respiration, temperature, nor muscular action, were materially affected. This preparation is mentioned by Dr. Farre, in his work on the "Malformations of the Human Heart;" and a detailed description of it, accompanied by two figures, is given in the ninety-fifth volume of the *Philosophical Transactions*, at page 228.—*London Lancet.*

